# Circulating redox status in sheep naturally infected with *Trichophyton verrucosum*

**DOI:** 10.1007/s11250-022-03284-7

**Published:** 2022-09-10

**Authors:** Mostafa A. Saleh, M. H. Rateb, Elham A. Abd-Allah, Ghada A. E. Mohamed

**Affiliations:** 1 Biochemistry Unit, Regional Animal Health Research Laboratory, Animal Health Research Institute, Agriculture Research Center, Assiut, 71526 Egypt; 2 Department of Zoology, Faculty of Science, New Valley University, El-Kharga, 725211 Egypt

**Keywords:** Oxidative stress, Sheep, *Trichophyton verrucosum*

## Abstract

*Trichophyton verrucosum* is a zoophilic dermatophyte that causes skin inflammation. The present study aimed to evaluate the redox status in the blood of sheep clinically infected with *T. verrucosum*. According to clinical and mycological investigations, 48 juvenile male Balady sheep were selected in their natural habitat and divided into four groups depending on the lesion size: mild (MID), moderate (MOD), severe (SEV) *T. verrucosum* infection, and healthy control groups. Compared to the controls, plasma superoxide anion increased (*P* < 0.05) in both MOD and SEV but total peroxides (TPx) gradually increased (*P* < 0.05) in MID followed by MOD and SEV. Superoxide dismutase and total antioxidant capacity (TAC) were higher (*P* < 0.05) in MID and lower (*P* < 0.05) in MOD and SEV than in controls, but SEV showed lower TAC than MOD. Malondialdehyde (MDA, a lipid peroxide marker) increased (*P* < 0.05) in SEV than in controls, but protein carbonyl (PC, a protein peroxidation marker) was augmented (*P* < 0.05) as lesions progressed from mild to severe. The oxidative stress index (TPx/TAC ratio) progressively increased (*P* < 0.05) in MOD and SEV. The correlation of PC was positive with TPx and negative with TAC (*P* < 0.01). In conclusion, sheep infection with *T. verrucosum* is characterized by increased TPx and decreased TAC in plasma depending on the lesion area. The redox status is shifted towards the oxidizing state, particularly in MOD and SEV cases. This results in a condition of OS, which may contribute to the pathogenesis of the disease.

## Introduction

Trichophyton verrucosum is a zoophilic dermatophyte that causes skin inflammation. Although the disease seems to be superficial, it can induce life-threatening systemic reactions in humans as well as economic losses in farm animals, particularly in the Middle East and North Africa region (Osman et al. 2021). *T. verrucosum* is a host-specific dermatophyte essentially adapted to cattle and less frequent in small ruminants (Carter 1990; Gnat et al. 2019a). However, outbreaks of *T. verrucosum*-induced trichophytosis can occur in sheep herds, resulting in exudative dermatitis with a severe inflammatory response (Sargison et al. 2002). In Egypt, the mixed rearing of small ruminants with cattle is responsible for the spread of *T. verrucosum* among animals and humans (El-Tras et al. 2015).

The diverse dermatophyte-host interaction is a key player in the pathogenesis of the disease (Gnat et al. 2019b). *T. verrucosum* secretes proteolytic keratinases that have the highest activity in sheep when compared to other species and plays an important role in the pathogenesis of the infection by generating inflammatory reactions (Lepper 1974; Martinez-Rossi et al. 2017; Gnat et al. 2019a, b a, b).

In addition to local inflammatory responses, *T. verrucosum* infection induces systemic reactions in the form of reticuloendothelial stimulation accompanied by neutrophil and mononuclear cell activation (Lepper 1974; Heinen et al. 2017). As a result, highly toxic reactive oxygen species (ROS), particularly superoxide anion (O֗_2_¯), are enhanced to act as a host defense against the invading pathogen (Lord and Vyas 2019). The body, on the other hand, has a variety of potent enzymatic and nonenzymatic antioxidants that work in tandem to scavenge and neutralize the enhanced ROS (Portugal et al. 2007; Beigh et al. 2014). According to Erel (2005), the cumulative action of these antioxidants is known as total antioxidant capacity (TAC).

Unfortunately, excessive ROS generation can overcome the antioxidant power with a subsequent shifting of the redox equilibrium towards the prooxidative state (Halliwell and Gutteridge 2015). In such conditions, the cytotoxic peroxides are increased and the oxidative stress (OS) state is enhanced (Sies et al. 2017). The triggered OS can induce oxidative damage to lipids, especially polyunsaturated fatty acids, to form lipid peroxides, and can also oxidize the side chains of amino acid residues in proteins to form protein carbonyls (Valacchi et al. 2018).

Oxidative stress appears to play a role in the pathogenesis of dermatophytosis in both human and experimental studies (Martinez-Rossi et al. 2017; Barygina et al. 2019). Dermatophytosis-induced OS has also been observed in cattle (Al-Qudah et al. 2010) and dogs (Beigh et al. 2014). The ratio of TOS to TAC was proposed as a method for determination of the oxidative stress index (OSI) that could be used to examine the degree of OS status (Sánchez-Rodríguez and Mendoza-Núñez 2019).

The host–pathogen interaction in dermatophytosis is considered an old unfinished story (Yoshikawa and De Almeida 2017) and is still poorly delineated (Lord and Vyas 2019). A full understanding of the pathogenesis of the infection may provide the venue for disease control. To gain a deeper understanding of this issue, the present study aimed to evaluate the redox and oxidative stress status in the blood of sheep clinically infected with the dermatophyte *T. verrucosum*.

## Materials and methods

### Animals

The present study included 48 juvenile male Balady sheep (5.5 ± 0.3 months of age). These animals were selected from sheep reared in the eastern part of Assiut governorate, Egypt. Clinical and mycological investigations proved that 36 sheep were infected by *T. verrucosum*. The remainder 12 sheep were clinically and mycologically healthy. The selected sheep have been reared similarly in small flocks under an un-organized farming system without satisfactory standards of feeding and management. During the day, these animals in small herds were collected and confined by their owners to limited areas for grazing pastures, agricultural wastes, and crop residues. In the afternoon, Berseem Hegazi (*Medicago sativa*) was offered indoors without extra supplementation. Infected sheep had a history of dermatological lesions for 1.5–3 months before the examination. Both the infected and control sheep had no history of recent topical or systemic medications for at least 1 month. The body condition score was visually evaluated in all sheep according to Kenyon et al. (2014). The score was performed by the same observer and ranged from 1 to 5 (1 = emaciated; 5 = obese) with an interval of 0.5 point. Samples from feces, blood, and skin scrapings were subjected to parasitological examinations to confirm that the selected sheep were free from internal or blood parasites in addition to external parasites specially mange mite infestation. Healthy sheep were negative for threefold KOH examinations and fungal cultures. Lesions in the infected sheep were confined only to the head and ears.

The selected sheep were divided clinically into four groups according to the lesion size. Only one of the authors evaluated these lesions. The first group showed 1–2 lesions, each with an approximate diameter of 2–4 cm (*n* = 14, mild infection, MID). The second had larger lesions that covered about a quarter to a third of the head and ear (*n* = 12, moderate infection, MOD). Animals with lesions that covered the majority of the head and ears were considered to have a severe infection (*n* = 10, SEV). The remainder (*n* = 12) were healthy sheep and were used as a control group.

### Blood sampling

In the early morning, blood was sampled from the jugular vein in 10-ml heparinized vacuum tubes and sent immediately to the laboratory on crushed ice. After centrifugation (at 2500 × g for 15 min at 4 °C), clear non-hemolyzed plasma was separated and stored at − 80 °C until analysis within a week.

### Mycological investigations

Skin lesions were cleaned with 70% ethyl alcohol. The margins were scraped in a sterile Petri dish to collect hair and skin tissues. In healthy sheep, areas around the muzzle and eyes, in addition to the ears, were also sampled. The scraping material was divided into two portions. The first was digested with 10% KOH for 15 min and examined for the presence of spores and hyphae under a microscope. The rest of the specimen was inoculated into Sabouraud’s dextrose agar supplemented with chloramphenicol and cycloheximide (Mycobios Selective Medium; Oxoid, Basingstoke, UK). The medium was enriched with inositol and thiamin and incubated at 28 °C and 37 °C for 2–6 weeks. Incubation was checked 2–3 times a week. A lactophenol cotton blue preparation was made for morphological studies of the isolates (Rebell and Taplin 1974).

### Biochemical analysis

#### Superoxide anion (O֗_2_¯)

As a major component of free radicals, O֗_2_¯ was measured according to the procedure of Podczasy and Wei (1988), which is based on the reduction of the tetrazolium compound, p-iodonitrotetrazolium (INT) by O֗_2_¯ generated by xanthine/xanthine oxidase to a water-soluble product (reddish pink) and the optical density is read at 505 nm.

#### Total peroxides (TPx)

Measurement of total peroxides or so-called reactive oxygen metabolites (ROM) is usually used as an indirect method to estimate the reactive oxygen species (ROS) concentration (Erel 2005). Plasma TPx concentration was measured following the method described by Erel (2005). This method is based on the oxidation of Fe^+2^ (as a catalyst) to Fe^+3^ by the various types of peroxides in the presence of xylenol orange in an acidic medium, which binds with Fe^+3^ to form a colored complex whose optical density is read at 560 nm.

#### Total antioxidant capacity (TAC)

Total antioxidant capacity (TAC) was determined by using the colorimetric ABTS assay kit (Beyotime Institute of Biotechnology, Haimen, China) according to the method described by Miller et al. (1993). In this method, the sample antioxidants inhibit peroxidase-mediated oxidation of 2,2-azino-bis-(3-ethylbenzothiazoline-6-sulfonate, colorless solution) to the radical cation (blue-green). TAC was expressed as mmol Trolox equiv/L.

#### Superoxide dismutase (SOD)

The activity of SOD was estimated according to the method described by Misra and Fridovich (1972). In an alkaline medium, SOD inhibits the autoxidation of epinephrine to adrenochrome. OD was measured at 480 nm.

#### Lipid peroxide (malondialdehyde; MDA)

Lipid peroxidation was determined as thiobarbituric acid reactive substances (TBARS) according to Placer et al. (1966). The method depends on the formation of a color complex between the products of lipid peroxidation and thiobarbituric acid (TBA). In this assay, 1,1,3,3-tetramethoxypropane was used as a standard. The absorbance was read at 548 nm against bi-distilled water as a blank.

#### Protein oxidation (protein carbonyls; PC)

Protein carbonyls were measured by using DNPH (2,4-dinitrophenylhydrazine) according to the method of Levine et al. (1990). In brief, 10 μl of plasma was incubated with 1 ml of 0.2% DNPH (dissolved in 2.5 M HCl) for 1 h. Then 1 ml of 20% trichloroacetic acid was added to precipitate the protein. After shaking and centrifugation (3000 × g for 10 min), the supernatant was decanted and the pellets were washed three times with 1 ml of ethanol and ethyl acetate solution (1:1; vol./vol.). The final pellet was re-dissolved in 1.5 ml of 6 M guanidine hydrochloride and the color of the supernatant was read at 370 nm.

#### Oxidative stress index (OSI)

The TAC unit, mmol Trolox equiv/L, was converted to µmol Trolox equivalent/L. The OSI value (as an indicator of the degree of oxidative stress) was determined as the percentage ratio of TPx to TAS as follows: OSI (Arbitrary unit) = (TPx, µmol/L / TAC, µmol Trolox equv/L) × 100 (Sánchez-Rodríguez and Mendoza-Núñez 2019).

#### Statistical analysis

The packaged SPSS program for Windows version 10.0.1 (SPSS, Chicago, IL, USA) was used for statistical analysis. The normal distribution of data for each parameter was checked using the Shapiro–Wilk test. The data were analyzed using a one-way analysis of variance (ANOVA) and expressed as mean ± standard error (SE). Differences between groups were determined using pair-wise multiple comparison procedures (when a significant F test was found) using Duncan’s new multiple range test. The significance level was set at *P* < 0.05. Linear regression analysis (*R*^2^) and Pearson’s correlation (*r*) were done on the paired data obtained by the individual cases.

## Results

No systemic reactions were observed in the infected animals. Those with extensive lesions showed variable degrees of ill-thriftiness and a rough coat. Weight differences between the control (32.17 ± 0.44 kg), MID (32.08 ± 0.46 kg), and MOD (31.67 ± 0.48 kg) groups were not significant (*P* > 0.05), but the SEV (30.97 ± 0.57 kg) tended to be lower (*P* = 0.051) than the control group. There were no apparent differences in the body condition score between all groups. The average score values recorded in all animals ranged from 2.5 to 3.0 units.

**Fig. 1:**
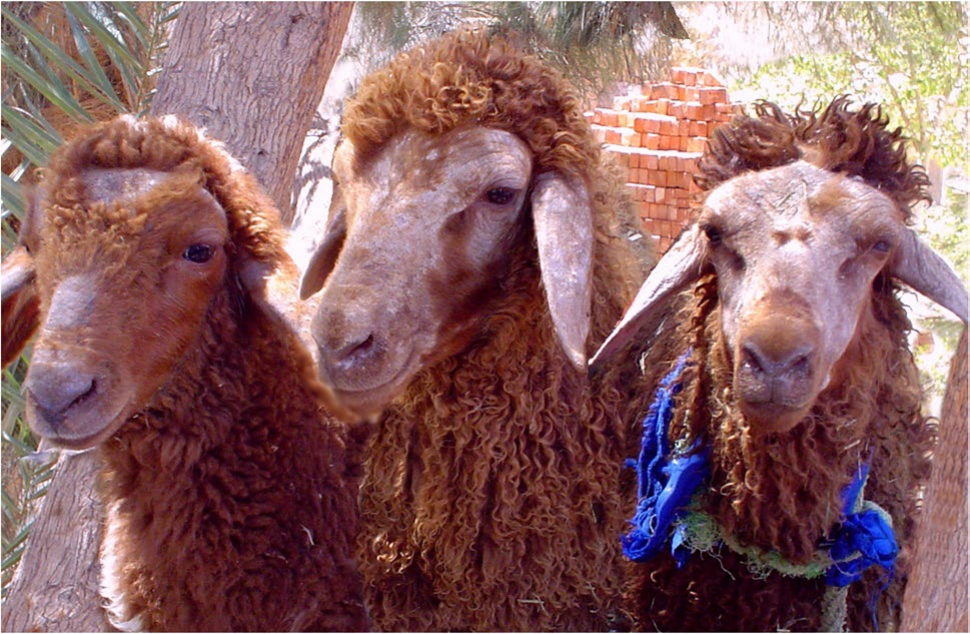
Clinical signs of T. *verrucosum* infection in sheep. Lesions are manifested by defined circumscribed areas of scaly alopecia with grayish wart-like crusty lamellar scales. Lesions may coalesce forming strange exfoliated configurations covering most of the head and ear pinna

Lesions tended to be non-pruritic in most of the infected animals, but only a mild pruritic reaction was seen in three of the extensively infected animals, where a gentle rubbing of the head and ear against hard objects was observed. Lesions were confined only to the head and dorsum of the ear pinna. Fleece and other parts of the body were not affected. Lesions were manifested by defined, circumscribed areas of scaly alopecia with grayish wart-like crusty lamellar scales that were difficult to pull off the skin. At the rim, the active fresh lesions were erythematous. The surface below the crust was edematous, pink to red in color, exudative, and inflamed. These lesions were accompanied by necrotic areas with voluntary blood oozing and a fetid odor. Affected areas varied in diameter from circular areas of 2–4 cm to coalesced uneven or diffused areas, forming strange exfoliated configurations covering most of the head and ears (Fig. 1).

**Fig. 2:**
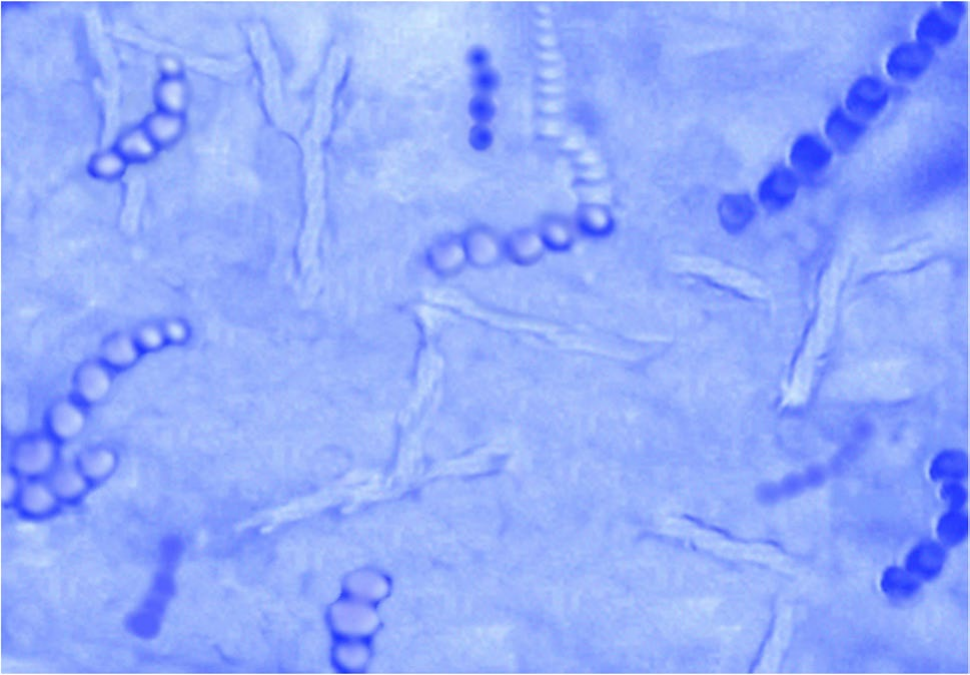
Chains of chlamydoconidia of *T. verrucosum* (lactophenol cotton blue, × 400)

Depending on mycological investigations and identification keys, the spores, hyphae, and typical colonies on the selective media were recorded. No other types of fungal infections were present, and the strain from the examined sheep was identified as *T. verrucosum*. Colonies grew slowly, taking 20–40 days after incubation at 37 °C. These colonies were button-shaped and white creamy in color with a velvety surface. Microscopically, the characteristic chains of thick-walled multi-septated chlamydoconidia with the string bean shape were seen with lactophenol cotton blue staining (Fig. 2).

**Fig. 3:**
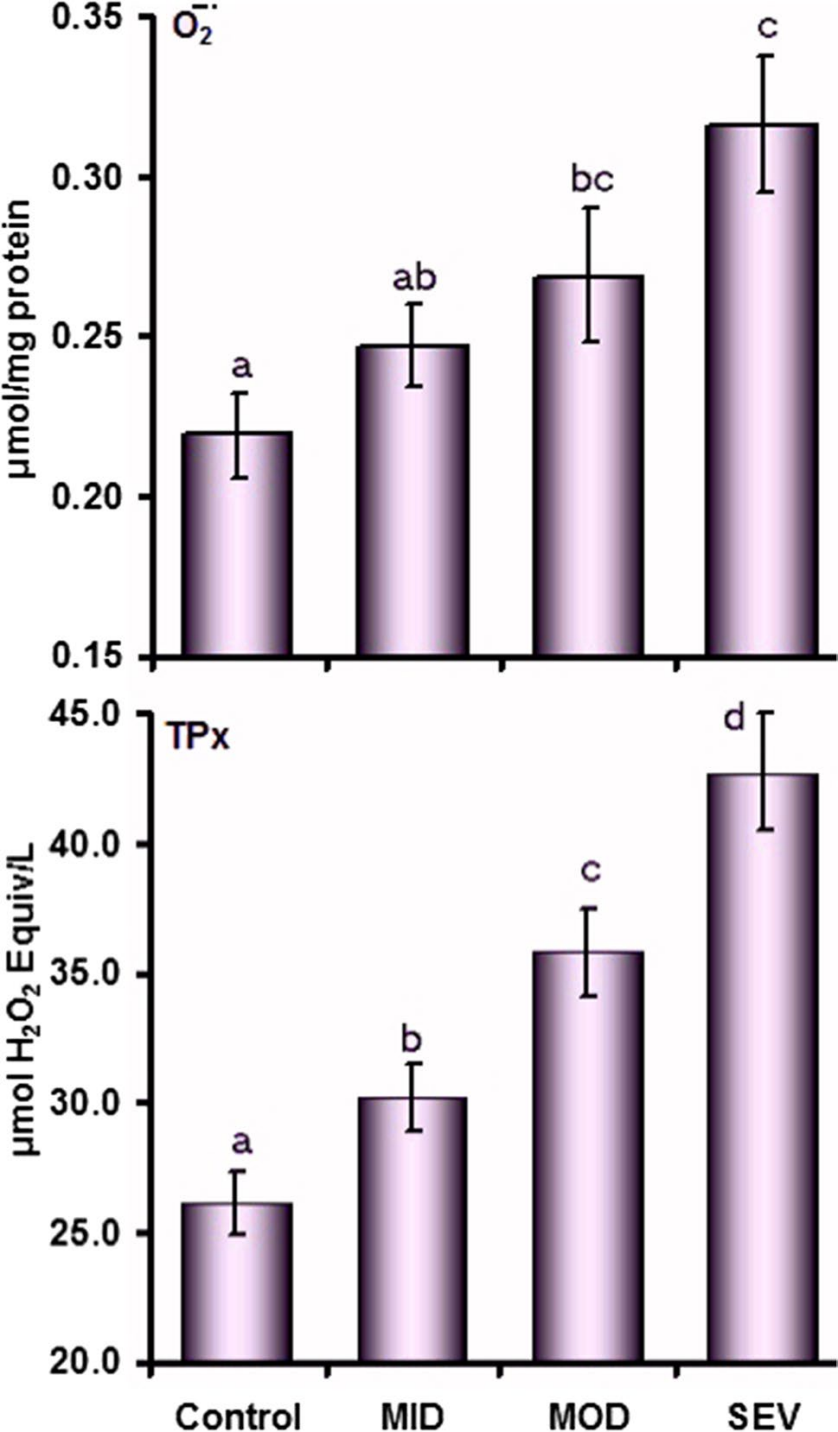
Plasma concentrations (mean ± SE) of superoxide anion (O֗_2_¯) and total peroxides (TPx) in control and mild (MID), moderate (MOD), and severe (SEV) dermatophytosis in sheep. ^a,b,c,d^Values with unlike descriptive superscript letters are significantly different (*P* < 0.05)

Concentrations of O֗_2_¯ did not change (*P* = 0.08) in the MID group but they increased in both MOD (*P* = 0.03) and SEV (*P* < 0.001) in comparison to the control values. Compared with the MID group, O֗_2_¯ value was higher (*P* = 0.008) in the SEV group. The values of TPx differed significantly (*P* < 0.05) between the four groups. There was a gradual increase in TPx concentrations in MID (*P* = 0.03), followed by MOD (*P* = 0.001) and SEV (*P* < 0.001) in comparison to the control group (Fig. 3).

**Fig. 4:**
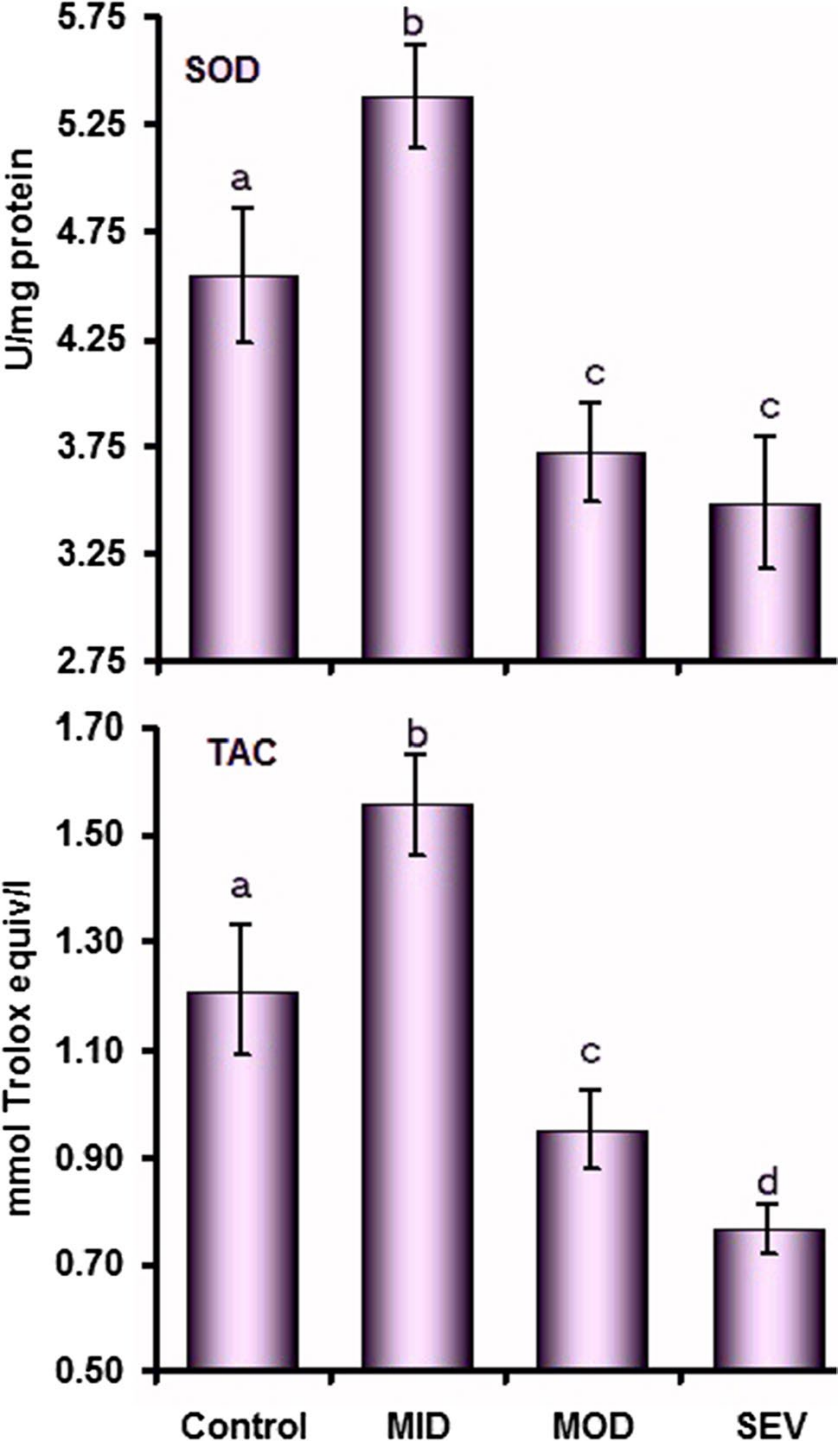
Plasma concentrations (mean ± SE) of superoxide dismutase (SOD) and total antioxidant capacity (TAC) in control and mild (MID), moderate (MOD), and severe (SEV) dermatophytosis in sheep. ^a,b,c,d^Values with unlike descriptive superscript letters are significantly different (*P* < 0.05)

The activity of SOD was higher (*P* = 0.02) in MID and lower in MOD (*P* = 0.03) and SEV (*P* = 0.02) in comparison with the control group. There was a non-significant difference (*P* = 0.27) between MOD and SEV. The same was observed for the TAC concentrations, where the values were higher in MID (*P* = 0.01) and lower in MOD (*P* = 0.03) and SEV (*P* = 0.004) in comparison with the control group. The SEV showed lower concentrations (*P* = 0.03) than the MOD group (Fig. 4).

**Fig. 5:**
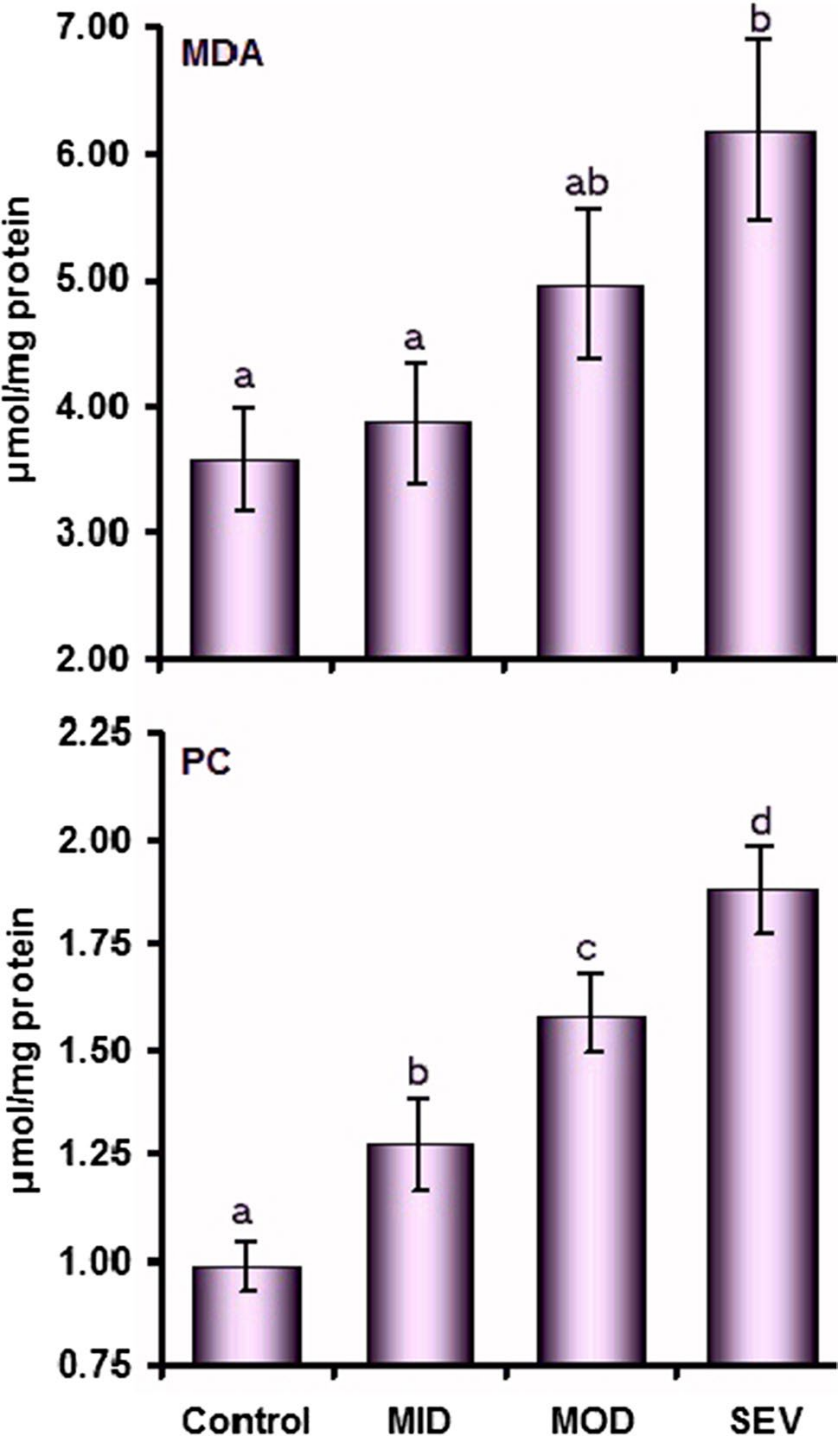
Plasma concentrations (mean ± SE) of malondialdehyde (MDA) and protein carbonyl (PC) in control and mild (MID), moderate (MOD), and severe (SEV) dermatophytosis in sheep. ^a,b,c,d^Values with unlike descriptive superscript letters are significantly different (*P* < 0.05)

Compared to the corresponding control values, concentrations of MDA did not change in both MID (*P* = 0.327) and MOD (*P* = 0.061) but increased (*P* = 0.003) in the SEV group. MDA values in MID and MOD did not differ (*P* = 0.097). The same was observed between SEV and MOD (*P* = 0.103), but SEV was higher (*P* = 0.01) than MID. On the other hand, there was a significant difference in PC concentrations between the four groups. PC augmented as lesions progressed from mild to severe. Its concentrations demonstrated a steady increase in MID (*P* = 0.014) followed by MOD (*P* < 0.001) and SEV (*P* < 0.001) compared to the controls (Fig. 5).

**Fig. 6:**
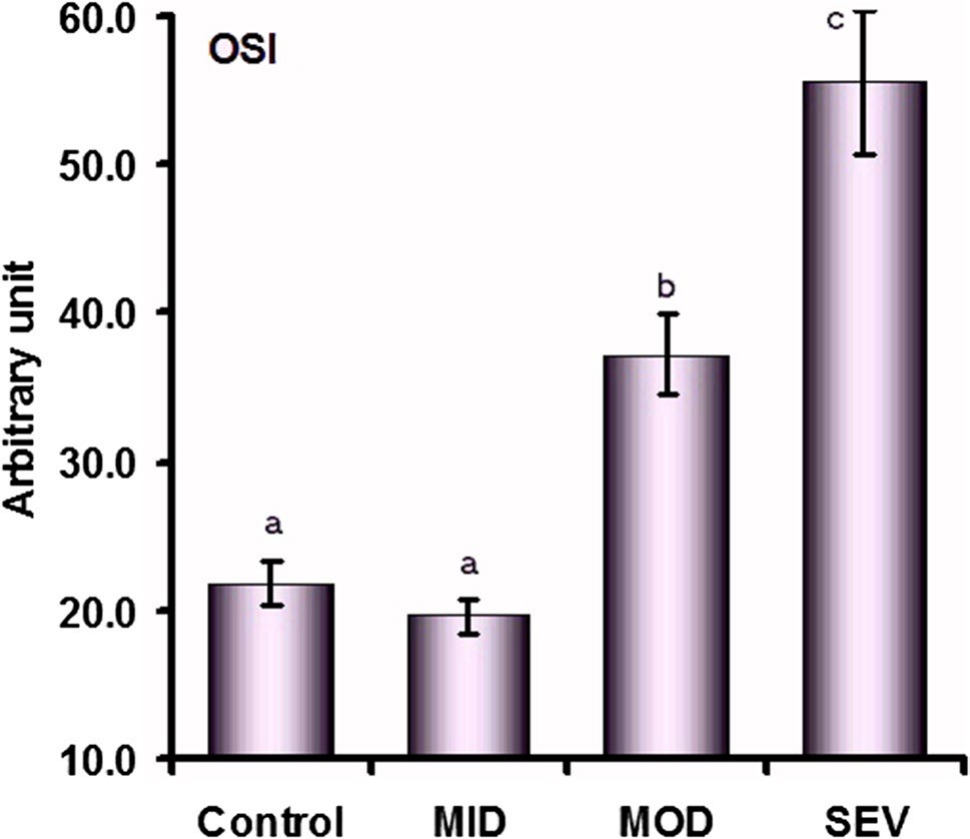
Oxidative stress index (OSI) value (mean ± SE) in control and mild (MID), moderate (MOD), and severe (SEV) dermatophytosis in sheep. ^a,b,c^Values with unlike descriptive superscript letters are significantly different (*P* < 0.05)

The oxidative stress index did not differ between MID and the control group (*P* = 0.089), but it progressively increased in MOD (*P* < 0.001) and SEV (*P* < 0.001), where the SEV group showed the highest value (Fig. 6).

**Fig. 7:**
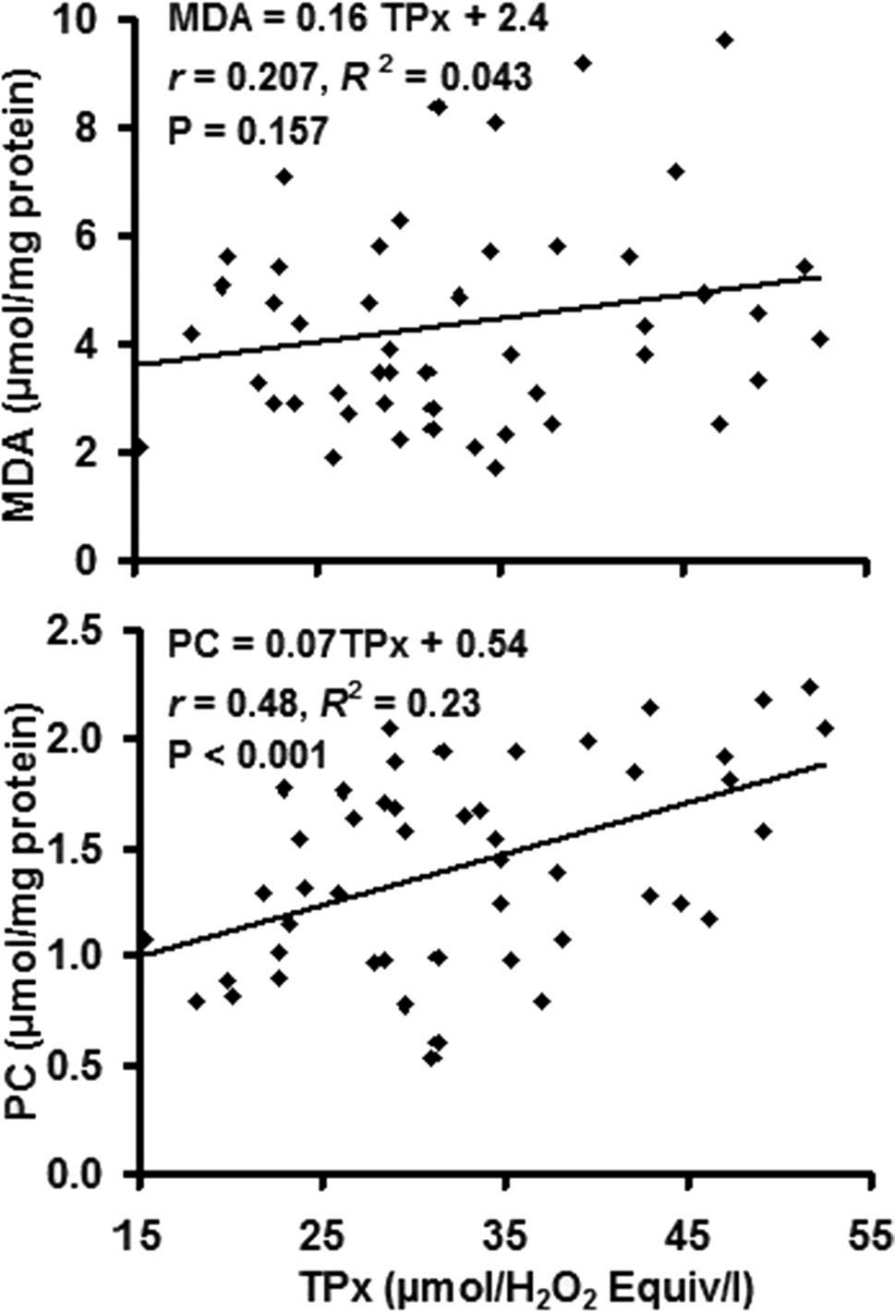
Correlation of total peroxides (TPx) with malondialdehyde (MDA) and protein carbonyl (PC) concentrations in sheep dermatophytosis

**Fig. 8:**
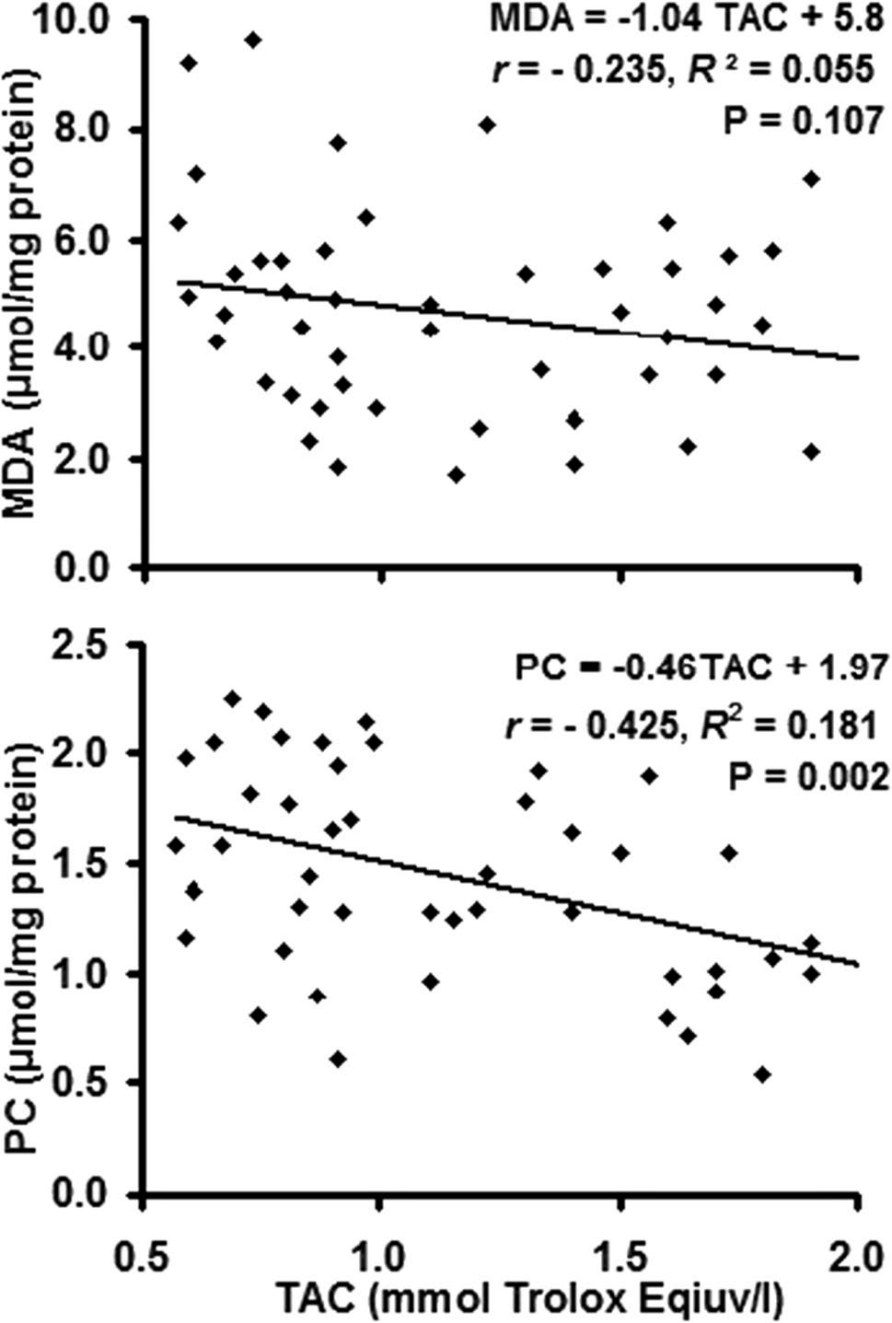
Correlation of total antioxidant capacity (TAC) with malondialdehyde (MDA) and protein carbonyl (PC) concentrations in sheep dermatophytosis

Linear regression analysis (*R*^2^) and Pearson’s correlation (*r*) of the paired data obtained by the individual cases (Figs. 7 and 8) revealed that TPx was not correlated with MDA (*P* = 0.157) whereas it was positively correlated with PC (*r* = 0.48, *R*^2^ = 0.23, *P* < 0.001). The same was observed for TAC, where it was not correlated with MDA (*P* = 0.107), but the correlation with PC was negative (*r* =  − 0.43, *R*^2^ = 0.18, *P* = 0.002).

## Discussion

The findings in this study provide reliable biochemical evidence for a circulatory redox imbalance in sheep infected with T. verrucosum. The redox status shifted towards an oxidizing state depending on the lesion size. This could be detected by the augmented TPx biomarkers and the accumulation of lipid and protein oxidative by-products coupled with inhibited TAC in MOD and SEV lesions. The present study is, to our knowledge, the first to describe the oxidative stress status in sheep with *T. verrucosum* infection.

Although *T. verrucosum* is well adapted to cattle, outbreaks with severe inflammation have occurred in sheep (Scott 1975; Sargison et al. 2002). Clinical findings of inflammatory reactions and exudative dermatitis recorded in the current study agree with those previously reported for sheep (Sargison et al. 2002; Roberson et al. 2012). Previous studies have shown that a dermatophyte can trigger a stronger inflammatory response in a non-adapted host than it does in its natural host (Heinen et al. 2017; Gnat et al. 2019b), which may explain the extensive and severe reactions observed in the investigated sheep in this study.

Despite the superficial localization of dermatophytosis, the systemic host-fungus interaction is still complicated and poorly understood. Infections with trichophyton species are limited to the hair and keratinized tissue of the epidermis, which makes them unique because they fail to penetrate the underlying tissues (Roberson et al. 2012). Pathogenic dermatophytes enhance the systemic immune response of the host, which restricts the infection to the superficial layers of skin (Heinen et al. 2017). Keratinases as proteolytic enzymes produced by *T. verrucosum* augment the activity of the non-professional coetaneous immune cells, epidermal keratinocytes, and dermal fibroblasts, and trigger the circulating neutrophils and monocytes (Lord and Vyas 2019). Gnat et al. (2019b) reported that keratinases correlate with the appearance of dermatophyte infections. As a result, pro-inflammatory cytokines such as interleukin-1α (IL-1α), IL-1β, IL-6, IL-8, and tumor necrosis factor-α are upregulated (Kabu and Sayin 2016; Hesse-Macabata et al. 2019). These cytokines can also be activated by the physical stimulation of the pathogen on the dermal dendritic cells (Kashem et al. 2015). Consequently, cascades of free radicals and ROS are generated (Calderon and Hay 1987; Lord and Vyas 2019; Barygina et al. 2019). Furthermore, the upregulated cytokines and ROS activate the pro-inflammatory nuclear factor-kappaB, which further amplifies free radicals and ROS generation (Sies et al. 2017). In the present study, the generation of O֗_2_¯ and TPx was enhanced progressively in mild, moderate, and severe infections, suggesting a link between the intensity of ROS generation and the increase in the circumference of infection by *T. verrucosum* on the skin of sheep. Our results agree with those reported for chronic dermatitis in humans (Grange et al. 2009; Zhang et al. 2019).

Despite the pathway variations of the different antioxidants, TAC represents the cumulative action of all of them (Halliwell and Gutteridge 2015). In this study, both SOD and TAC increased in mild infection and decreased with the increase of the infection area, becoming lower than that of the controls in moderate and severe infection. This might be expected when considering the chronicity of inflammation in *T. verrucosum* infection. Over time, the accumulation of ROS eventually exceeds the defense capacity of the antioxidant system (Bertino et al. 2020). In humans, Niwa et al. (2003) found increased SOD activity in mild dermatitis, but the activity was lower in severe than in moderate cases. Zhang et al. (2019) reported an association between the lower TAC and the severity of dermatitis in humans. In dogs with dermatophytosis, Beigh et al. (2014) found lowered functions of the antioxidants SOD, catalase, β-carotene, and vitamin C.

The generation of ROS is considered a formidable weapon against invading pathogens and powerful fungicidal tools, but they are unspecific effectors and could severely damage the surrounding biological medium (Colitti et al. 2019). Lipids, especially polyunsaturated fatty acids and the side chains of amino acid residues of proteins, are sensitive to oxidation by the action of free radicals. According to Halliwell and Gutteridge (2015), oxidation of these molecules results in the formation of high molecular weight aggregates that are resistant to degradation and are accumulated as damaged and toxic substances. Malondialdehyde (MDA) as a biomarker of lipid peroxidation and protein carbonyls (PCs) as a biomarker of protein oxidation have attracted substantial attention for their roles as pro-oxidants, toxic end-products, and a universal index of oxidative stress in skin diseases (Mori et al. 2017; Sies et al. 2017). The enhanced MDA and PC generation shown in this study reflects a respective augmentation of lipid peroxides and protein oxidative by-products in the blood of sheep with *T. verrucosum* infection. This provides further evidence of enhancement of the pro-oxidants and the generation of toxic molecules, which might be key in understanding the mechanisms of *T. verrucosum* infection in sheep.

In the present study, plasma MDA increased in moderate and severe cases, but PC progressively increased in mild, moderate, and severe infections. In addition, PC was positively correlated with TPx and negatively correlated with TAC, but MDA did not show significant correlations with TPx or TAC. These results suggest that protein oxidative damage is continuously occurring and progressively amplified as the lesion area increases. Furthermore, the obtained results suggest that the increased PC concentrations are linked to the increased prooxidant power and the decreased antioxidant potential. Previous reports showed that mild conditions of oxidative stress are characterized by higher oxidized protein concentrations (Valacchi et al. 2018). Furthermore, PC has a major advantage over MDA as a marker of oxidative damage because it is early formed and has considerably longer half-lives compared with MDA (Halliwell and Gutteridge 2015). Earlier studies showed that the level of protein carbonyl was elevated and correlated directly with the severity of human dermatitis (Niwa et al. 2003). A positive correlation of the high levels of MDA and PC with the severity of dermal diseases was shown in humans (Nakai et al. 2009; Yazici et al. 2016) and in dogs (Kapun et al. 2012). A negative correlation of MDA with the antioxidants β-carotene, SOD, and catalase was noticed in dermatophytosis-affected dogs (Beigh et al. 2014). It seems that these responses differ according to the host species, environmental conditions, and level of nutrition, in addition to the virulence of the invading dermatophyte and duration of the disease (Gnat et al. 2019b).

Oxidative stress can result from increased TPx, decreased TAC, or even both, so the main players in redox homeostasis are TPx and TAC (Valacchi et al. 2018). The OSI provides an objective judgment of the pro-oxidant/antioxidant interactions not observed by the separate investigations of both components (Sánchez-Rodríguez and Mendoza-Núñez 2019). Baek and Lee (2016) suggested that OSI could provide useful information on OS and redox status in chronic skin diseases. Our results suggest the participation of OS challenge, especially in moderate and severe infections. In these cases, the redox status shifted towards an oxidizing state, where TPx increased and TAC decreased. This also implies that dermatophytosis in sheep is likely to extend far beyond local effects on the skin and play an integral role in host systemic reactions. On the other hand, OS markers change in the same direction in blood and tissues (Veskoukis et al. 2009; Margaritelis et al. 2015). Therefore, blood concentrations of these markers may reflect corresponding changes in the affected skin.

## Conclusion

Sheep infection with *T. verrucosum* is characterized by increased TPx and decreased TAC in plasma depending on the lesion area. The redox status is shifted towards the oxidizing state, especially in MOD and SEV cases. This results in a condition of OS, which may contribute to the pathogenesis of the disease.

## Data Availability

The datasets and materials are available from the corresponding author on reasonable request.
